# Could Physical Activity Have any Role in Cardiovascular Disease Prevention in Prisoners? A Systematic Review

**DOI:** 10.3390/ijerph18052307

**Published:** 2021-02-26

**Authors:** Veronica Papa, Domenico Tafuri, Mauro Vaccarezza

**Affiliations:** 1 Department of Motor Sciences and Wellness, University of Naples “Parthenope”, 80132 Napoli, Italy; domenico.tafuri@uniparthenope.it; 2 FAPAB Research Center, 96012 Avola, Italy; 3 Curtin Medical School & Curtin Health Innovation Research Institute (CHIRI), Curtin University, Kent St., Bentley 6102 WA, Australia; mauro.vaccarezza@curtin.edu.au

**Keywords:** CVD, physical activity, prevention, prison

## Abstract

More than 10.74 million people are currently held in penal institutions worldwide. Moreover, there is also evidence that the percentage of elder and female prisoners has been consistently growing. Cardiovascular diseases are the leading cause of death worldwide. Exercise training and physical activity help to prevent both primary and secondary cardiovascular events. Data on the influence of physical activity on the well-being in prison population is scarce. Here, we discussed, in a systematic review, the general health conditions and the cardiovascular risk profile in the prisoners compared to the general population and evaluated whether or not exercise could be a valuable tool in preventing these diseases in inmates. We performed a systematic review following the Preferred Reporting Items for Systematic Reviews and Meta-Analyses (PRISMA) statement: 769 were initially identified, and a total of 24 studies were finally included. Nine studies evaluated the health conditions in prisoners, five studies evaluated the incidence of cardiovascular disease (CVD) and coronary heart disease (CHD) in the prison population, and 10 studies evaluated the feasibility and the effectiveness of exercise programs in prisoners. Sports-educational programs can benefit prison inmates. It appears that supervised exercise training is an effective coping strategy to deal with incarceration. Moreover, it seems the sports programs might be a useful tool in improving physical and mental health of prisoners as well as in decreasing cardiovascular risk factors.

## 1. Introduction 

A growing body of scientific evidence, as well as the World Health Organization (WHO), recently ranked cardiovascular diseases (CVDs) as the leading cause of death worldwide, defining them as a global concern, given the high percentage of deaths in several countries [1].

CVD has multifactorial causes that are related to non-modifiable (family history) and modifiable risk factors (hypertension, inadequate diet, abusive consumption of alcohol and other psychoactive substances, dyslipidemia, obesity, sedentary lifestyle, among others) [1].

More people die annually from CVDs than from any other cause. In 2016, 17.9 million people died from CVDs. CVD represents 31% of all deaths; moreover, heart attack and stroke are the leading cause of death from CVD [2].WHO also reported that around 55% of the adult population were physically inactive. This percentage likely increases among the prison population. 

In a recent paper, Lennon and coworkers [3] reviewed the definition of CVD, their associated risk factors, and global health impact. CVD have multiple synergistic causes that require a global approach to their diagnosis and treatment. In this review, the authors defined and classified risk factors into three main groups: modifiable, maybe modifiable, and non-modifiable risk factors.

Likely, modifiable risk factors include education level and poverty status. Data collected from the prison population in the United States, which leads the world in the number of individuals under correctional supervision, clearly demonstrated that ethnic minority as well as low-income groups have higher risk of incarceration [4,5]. Moreover, low education level and poverty status might affect frequency of incarceration as well.

Modifiable risk factors include tobacco use, obesity or overweight, sedentary lifestyle, poor diet, and excessive alcohol consumption, which, unfortunately, are common habits in inmates [6].

According to Sanchez-Lastra and coworkers [7] and previously Arries and Maposa [8], incarceration leads to inequalities in disease burden and an increased risk for CVD

Detention seems to be associated with stress, hypertension, and other factors related to the pathogenesis of CVD. Furthermore, according to the same authors, physical inactivity has a higher incidence in prisoners compared to the general population. Additionally, Leuzzi and coworkers recently demonstrated that the prevalence of heart failure approximately doubles with each decade of life; due to dramatic increases in the aging population, the heart failure epidemic will grow significantly in the coming decades [9].

It is now well established that high levels of physical activity (PA) are associated with fewer CVD events. The mechanisms underlying this inverse association are still unclear. Recent evidence demonstrated that differences in modifiable cardiovascular risk factors might mediate this effect, significantly modulating inflammatory signaling pathways involved in atherosclerosis. Moreover, exercise training is a potent stimulus that could control and decrease primary and secondary cardiovascular events [2,10,11]. It is now clear that it is possible to prevent most cardiovascular diseases by addressing behavioral risk factors such as tobacco use, unhealthy diet and obesity, physical inactivity, and harmful alcohol habit using populatio{Citation}n-wide strategies. Concerning the special population examined in our paper, according to the World Prison Population List, in 2018, more than 10.74 million people are held in penal institutions throughout the world. Moreover, according to the National Corrections Reporting Program (USA) [12], the percentage of elders (aged 55 or older) and women has been consistently growing and surpassed the number of young adults aged 18 to 24 years old [12].

Morris, in 1961, appears to be the first to demonstrate a correlation between PA and the risk of cardiovascular disease [13]. Since then, many studies have evaluated and confirmed the inverse association between exercises and CVD, further demonstrating that PA reduces CVD risk [14,15].

However, how PA decreases CVD risk is not well understood [16]. Tinken and coworkers [10] were the first to investigate the protective effects of vascular remodeling during exercise in healthy subjects. They demonstrated that shear stress and vascular remodeling played a crucial role in the benefits of exercise in CVD. Atherosclerosis and inflammation seem to be the cross-talking pathways between the two diseases [17], the shear stress and the remodeling of vascular endothelium being the common process both in CVD and inflammation.

So far, many studies have reported a significant inverse association of PA with mortality and CVD morbidity. Mora and coworkers in 2007 [14] analyzed the baseline levels of hemoglobin, lipids, creatinine, and inflammatory/hemostatic biomarkers of 27,055 healthy women. They also evaluated women’s self-reported PA, weight, height, hypertension, and diabetes and further demonstrated that CVD’s risk decreased linearly with higher PA levels. In particular, inflammatory/hemostatic biomarkers seemed to have higher relevance to lower risk (32.6%), followed by blood pressure (27.1%).

The regular physical exercise decreased risk of developing a chronic illness (such as cardiovascular disease, osteoarthritis, diabetes, and depression) and premature death. Exercise’s beneficial effect is associated with increased physical fitness, muscular strength, and decreased adiposity [16]. 

In 2012, Li and Siegrist [18], in a systematic review and meta-analysis, analyzed the correlations between PA and cardiovascular risks. The authors analyzed occupational PA and leisure time PA and distinguished coronary heart disease (CHD) and stroke, respectively. Their results suggested that high level of leisure time physical activity has a beneficial effect on cardiovascular health by reducing the overall risk of incident CHD and stroke among men and women by 20 to 30 percent, while moderate level of occupational physical activity might reduce 10 to 20 percent risk of CVD. 

Prisoners, both younger and older, reported musculoskeletal problems, probably caused by physical inactivity. Over the past two decades, national and international guidelines recommend PA’s practice in a non-specific manner but preferably recommended if carried out as a sport to reduce musculoskeletal problems (such as back pain) related to physical inactivity [18,19,20]. Moreover, the positive relationship of exercise with psychological well-being has also been established [21,22]. Of note, Cashin and coworkers [23] proposed that muscle atrophy might be associated with physical inactivity and many other chronic illnesses, and therefore increasing and strengthening muscle might have protective effects in chronic diseases and cognitive decline.

In 2012, Gersh et al. [24] were the first to quantify the dose-response relation between PA and coronary heart disease (CHD) risk: they performed a meta-analysis of cohort studies investigating PA and primary prevention of CHD, published in English since 1995. In accordance with the international guideline, the authors demonstrated that people who met the basic US physical activity guideline had a 14% lower risk of CHD than those with no leisure-time PA. Furthermore, people who met the advanced guidelines had a 20% lower risk of CHD. They also reported a lower relative risk among physically active persons below the basic guideline. Gersh and coworkers supported the guideline’s assertion that some PA is better than none.

Interestingly, they observed significant sex/gender-related differences in cardiovascular disease; PA’s dose-response effect is such that CHD’s association seems stronger in women than in men.

More recently, in a systematic review and meta-analysis, Lin and colleagues demonstrated that exercise training improved cardiorespiratory and cardio-metabolic biomarkers, including lipid metabolism, insulin resistance, and systemic hemostasis/inflammation [25]. 

Although it is now well established that regular PA yields significant health benefits, current guidelines on physical activity mainly target middle-aged adults. There is less evidence demonstrating the effectiveness of physical activity in increasing cardiovascular health in older adults. In a recent paper, Lachman and coworkers [26] compared the association between PA and CVD risk levels in middle-aged to elderly individuals. They analyzed 24,502 study participants aged 39–79 years from the EPIC Norfolk prospective population study. They demonstrated that the inverse association between PA and CVD risk was significant in the elderly and comparable with middle-aged individuals. In addition, they also observed that modest levels of PA granted some benefits in terms of CVD risk, compared to being completely inactive.

Recent findings also indicated that mental disorders might share a CVD relationship, particularly CHD [27,28].

According to Walker and coworkers, who in a recent study analyzed the mortality rates in mental disorders, up to 14.3% of all deaths worldwide (approximately eight million) each year might be linked to mental disorders [29].

Prisoners’ most frequent conditions with higher levels than the general population are mental illness, depression, infectious diseases, and other non-communicable diseases potentially involved in CVD such as hypertension, diabetes, and musculoskeletal problems [30]. Of note, the incidence of these diseases is usually higher in the prison population in comparison to the general population [30].

In terms of the general population, more than 300 million people suffer from depression worldwide. Therefore, it is likely that this disease might become one of the leading causes of disability worldwide by the next ten years [31,32].

The connection between the two pathologies is in terms of etiology and biological, psychological, and genetic mechanisms: patients with CHD frequently suffer from depression and other mental illness; conversely, these mental disorders might correlate with a substantial increase in cardiovascular morbidity and mortality [28]. Although the association between depression and CVD has been extensively studied in patients suffering from existing CHD [31], the inverse relationship between them is now well established [33,34,35].

Mental illness and infectious diseases are the most typical conditions in prisoners; nevertheless, the risk of CVD increases more in inmates and prisoners than in the general population because of their unhealthy behaviors such as tobacco smoking, alcohol and drug use, high sugar diets, and physical inactivity that are likely to rise during imprisonment [6,36,37].

Data on the influence of PA on the well-being and quality of life in prison are scarce. Sports-educational programs have a beneficial effect on prison inmates regarding social development. Moreover, we do not know if PA and this socio-pedagogical function have the same potential in improving health parameters and deferring CVD in prisoners. Moreover, we review the health conditions in the prison’s population, the incidence of CVD and CHD, and whether or not exercise could be a valuable tool in the prevention of these diseases in prisoners.

## 2. Methods

**Figure 1:**
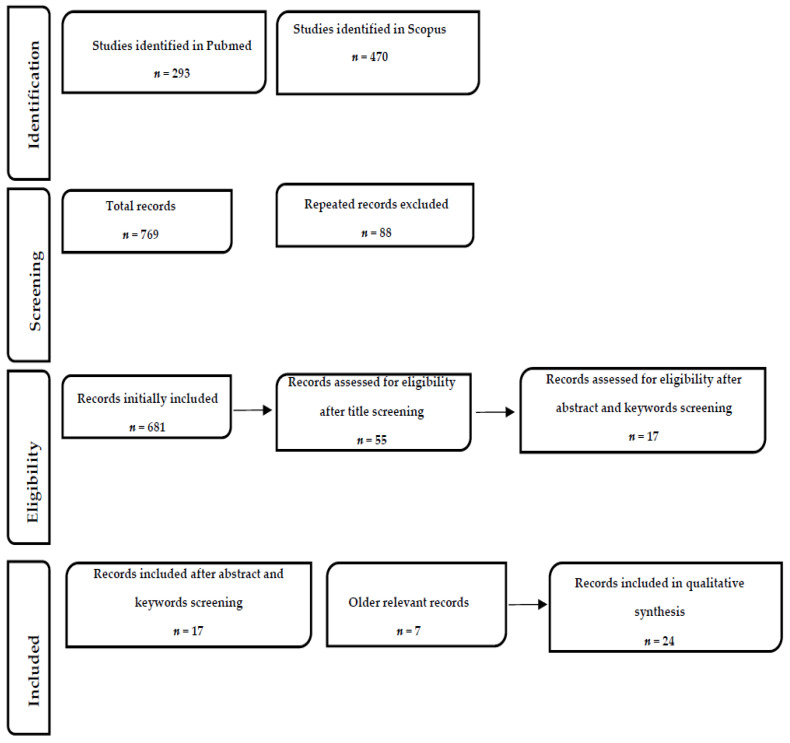
Flowchart of the reporting items for the systematic reviews (adapted the preferred reporting items for systematic reviews (PRISMA) statement.

To achieve the manuscript aims, we performed a systematic review following the Preferred Reporting Items for Systematic Reviews and Meta-Analyses (PRISMA). The search strategy was performed between July and December, 2020. The terms searched were (exercise) AND (jail OR prison OR prisoners OR inmate) as well as (CVD) AND (jail OR prison OR prisoners OR inmate). We selected only articles published in English and peer-reviewed journals and researched PubMed, Biomed Central, and Scopus. The inclusion criteria were any type of study focused on health and cardiovascular condition in prisoners, the effect of exercise in the prison population, and its association with any type of cardiovascular disease. To give high reliability to the data acquired, we preferred citations within the past ten years (2010–2020) but did not exclude often referenced, highly regarded older publications. We searched the reference lists of articles identified by this search strategy and selected additional publications that we deemed relevant. A total of 769 were initially identified. After duplicates removal, a total of 681 records were further processed. Title and keywords were used to identify or exclude records. Twenty-four studies were finally included (see Figure 1 for further details). For all articles, we analyzed abstracts and the full reference list. We decided to include only articles whose abstract unequivocally discusses the topic. The records included in the present study are listed in Table 1. All the authors agreed on the final number of studies included.

## 3. Results

According to the inclusion criteria, we screened 24 records. The records included in this study are listed in Table 1. We classified the studies according to the topic analyzed: health conditions in prisons, CVD and prisoners, and exercise in prisoners. Nine studies evaluated the health conditions in prisoners; five studies evaluated the incidence of CVD and CHD in the prison population. Finally, ten studies evaluated the feasibility and the effectiveness of exercise programs in prisoners. The main results in the studies analyzed are summarized in Table 2, Table 3 and Table 4, respectively. The three main topics are discussed below.

### 3.1. Health Conditions of the Incarcerated Population

According to Wang and coworkers [5] and Bonczar [4], in the last 30 years, both the number of incarcerated women and prisoners older than 55 dramatically increased, leading to a severe concern about the health care needs and cardiovascular risks in incarcerated individuals. Mental disorders, infectious diseases, cancer, and CVD are the primary health problem occurring in prisoners. Although randomized controlled trial evidence for prisoners’ mental health disorders is rare, the incidence of physical and psychiatric disorders is dramatically higher in prisoners than in the general population [6]. 

The first study investigating prisoners’ mental health dates back to 1918 and highlights many mentally ill people in Sing Sing prison in New York [53]. More recently, prisoners were at serious risk of suffering from psychosis and major depression and about ten times more likely to have an antisocial personality disorder than the general population [6,38]. 

In a recent study, Correll et al. [54] conducted the most extensive meta-analysis of CVD risk in people with severe mental illness (SMI); they analyzed 3,211,768 patients with SMI and 113,383,368 controls and demonstrated that SMI patients have a 50% increased risk of CHD versus controls. Moreover, in a systematic review, Fazel and Danesh previously analyzed data concerning mental disorders from 62 surveys of 23,000 prisoners and demonstrated the large prevalence of psychosis, personality disorder, and post-traumatic stress disorder in inmates [38].

In 2011, Fazel and Baillargeon [6] further demonstrated that the increased incidence of CVD and SMI in inmates is probably due to unhealthy lifestyles and behavioral and socio-economic factors, including higher drug rates, alcohol, and smoking use in prisoners compared to the general population. These factors increase the risk of infectious diseases, CVD, and oncological malignancies as well.

More recently, Wang and coworkers [5] reviewed the current status of the cardiovascular health of justice-involved populations and established that general risk factors in addition to incarceration-specific factors, such as exposure to the prison environment, poor diet, or lack of exercise, might increase the risk of CVD.

Surprisingly, a study from Condon et al.[39] showed that even when prisoners could exercise and improve their dietary regimes, they did not necessarily opt for them. Conversely, in 2007, Hinata and coworkers performed a retrospective analysis of 4385 medical charts of male prisoners in Fukushima Prison from 1998 to 2004, revealing 109 prisoners with type 2 diabetes. These patients’ follow-up demonstrated that well-regulated lifestyles such as regularly performing PA and healthy long-term diet habits might positively affect metabolic control in patients with type 2 diabetes whenever under correctional supervision [30].

Tuberculosis and chronic obstructive pulmonary diseases are frequently in inmates; in fact, the mortality rate for pulmonary diseases and tuberculosis dramatically increases in unemployed or unskilled manual employees, which are the occupations of most male prisoners aged 20 to 64 in the United Kingdom.

Finally, HIV/AIDS, as well as Hepatitis B and C infections in prisoners, is also a global health concern [30]. 

Interestingly, few studies have assessed the prevalence of non-communicable diseases in prisoners so far. A US survey showed higher age-adjusted rates of hypertension, diabetes, asthma, and arthritis in prisoners than in the general population [40]. 

Of note, although prisoners generally have major risk factors for cancer, US and Australian screening results reported similar yearly incidence rates in the general population and prisoners [6,41]. Moreover, convicted women seem to have a higher cancer rate than men: data on specific cancers suggest that cervical cancer is the most common in women, while skin cancer is the most common cancer in men [55]. 

In a more recent study, Munday and coworkers [42] analyzed the prevalence of non-communicable diseases in older people in prison. They quantitatively analyzed data from 93,862 participants aged 50 or over, reported across the 26 studies that met the inclusion criteria and covered 28 different non-communicable diseases. According to the data analyzed, it appears that the leading cause of morbidity and mortality are the same both in old prisoners as well as in the general population, the prevalence in convicted elder population is exceptionally high: 38% of older prisons had cardiovascular disease, 8% had been diagnosed with cancer, and diabetes estimates varied between 8% and 21%.

### 3.2. Impact of Cardiovascular Disease among Prisoners

In high-income countries, CVD is the most significant burden of disease. Moreover, its incidence is exceptionally high in lower socio-economic groups. Although CVD morbidity and mortality have declined over the past decades, the mortality rates attributable to CVD among incarcerated individuals continue to increase [56].

According to Packham and coworkers [43], almost 50% of prisoners’ deaths are due to natural causes. Between 2017 and 2019, the authors analyzed six male English prison populations to identify those eligible for a health-check and compare CVD risk data with those that were not. They identified that CVD risk was similar to community levels, but the prison population was ten years younger. Moreover, they further demonstrated that 21.8% of the prison population aged 35–74 already had comorbidities unsuitable for a health-check; of those that were eligible for the screening, a further 12.1% had a significant clinical risk for future CVD, and almost 40% suffered from depression or anxiety, further strengthening the case of need for good healthcare services in prison.

Interestingly, two recent studies fully elucidated the relationship between CVD and mental disorders [32,57], the most common health problem found in inmate populations.

De Hert and coworkers [28] demonstrated that several epidemiological data are consistent with people with the severe mental disease (schizophrenia, bipolar disorder, and major depressive disorder) having an increased risk of developing CHD, compared with control groups. Moreover, anxiety, intense stress, or post-traumatic stress disorder (PTSD) might also be associated with an increased risk of developing CHD. Mental diseases are common in patients with CHD and might be associated with a substantial increase in cardiovascular morbidity and mortality.

Dhar and Barton [57] explored the molecular mechanisms underlying the link between mental illness and CHD. Mental disorders can cause the deregulation of the sympathetic nervous system and hypothalamic–pituitary–adrenal axis; this, in turn, may have many downstream effects, including hypertension, left ventricular hypertrophy [58], coronary vasoconstriction, endothelial dysfunction [59,60], platelet activation, and the production of pro-inflammatory cytokines [61].

Moreover, the number of older adults in the criminal justice system is rapidly increasing worldwide. From 1996 to 2008, the number of inmates aged 55 or older increased 278% compared to a 53% increase in the overall jail population, and an estimated 500,000 older adults are incarcerated each year [44]. Therefore, it is now clear that the prisoners’ population is rapidly aging. This aging is a major daily health care problem for prison staff, since elderly inmates are among the most vulnerable of the prison population, both in terms of physical and mental health [6,45,62,63].

Recent data shows that how “older” is defined in criminal justice-involved individuals is extremely variable, ranging from 50 to 65 [64]; in a recent study, Greene and coworkers [44] compared 238 older jail inmates age 55 or older to 6871 older adults in the National Health and Retirement Study (HRS). They evaluated the socio-demographic differences and chronic and geriatric conditions, demonstrating that geriatric conditions are prevalent in older adults in jail at significantly younger ages than non-incarcerated older adults. In particular, they established that among older adults in jail with an average age of 59, the prevalence of several geriatric conditions was similar to that found among community-dwelling adults age 75 or older.

It is now well established that the incidence of CVDs increases with aging [17,65], although the majority of elderly patients suffering from CVD have either no conventional risk factor or just one risk factor [66,67]. 

In a pivotal study in 2001, Fazel [45] assessed the health of 203 men aged 60 or older in UK prisons. Prisoners rated their general health: 83% of elderly prisoners reported a long-standing illness or disability; of those, 71% reported CVD and 54% reported psychiatric disorders.

Wangmo et al. in 2016 [68] analyzed data from 380 male prisoners in Switzerland and described how inmates aged 50 or older suffered from more chronic diseases (hypertension, cardiovascular disease, diabetes) than younger inmates and that the number of diseases increased with age group.

In the last two decades, new evidence demonstrated sex/gender-related differences in CVD, highlighting the role of sex hormones in protecting women from CVDs and providing an advantage over men that is lost when women reach the menopause stage. This hormonal-dependent shift of sex-related CVD risk affects overall CVD epidemiology, particularly in light of the increasing trend of population aging [2]. Moreover, according to Plugge and coworkers [46], women in lower-income groups have higher CVD risk and report higher rates of cigarette smoking, lower consumption of fruit and vegetables, higher fat diet, and less total PA than women in higher-income or social groups. 

Recent findings also demonstrated how women now represent a larger proportion of the incarcerated population than before. In the last twenty years, the number of incarcerated women from low socio-economic status increased by more than 700% [5]. 

Previous research demonstrated that female prisoners have a greater incidence of mental health problems than male prisoners [69]; more recently, Caulfield demonstrated that, although women who reported mental health issues in prison experienced violence and mental disorders, a significant number of women reported first experiencing mental health and emotional problems only after entering custody [20]. 

Plugge and coworkers in 2009 [46] studied the short-term impact of imprisonment on the prevalence of five modifiable CVD risk factors (smoking, PA, diet, body mass index, and hypertension) in women prisoners on entry to prison and then one month after imprisonment. They analyzed modifiable CVD risk factors in 505 women prisoners on entry to prison and documented short-term changes in these risk factors one month after imprisonment. The results demonstrated that women prisoners were at high risk of CVD at the prison entrance, and after one month follow up, few improvements in risk factor could be detected. Women self-reported low levels of PA, with only 13.1% meeting the international recommendation for PA; of note, a comparison of PA levels before imprisonment and the following month demonstrated no significant improvements, and women remained relatively sedentary.

### 3.3. The Role of Physical Activity in the Prisoner’s Well-Being

Data on PA and quality of life in prisons are scarce. In 2008, Cashin and coworkers [23] analyzed the relationship between the levels of self-reported physical exercise and mental well-being, measured using the Beck Hopelessness Scale in a cohort of 914 prisoners within New South Wales. They established that, although weak, there was a statistically significant inverse correlation between total exercise time in minutes reported in prison per week and hopelessness; therefore, increasing exercise corresponded to a decreased reported hopelessness.

In 2014, Battaglia and coworkers estimated which kind of physical activity could better improve inmates’ health status and fitness levels. They measured the effectiveness of a nine-month intervention program of physical activity on the psychological welfare of 64 prisoners. Participants were included and randomly assigned to three groups: cardiovascular plus resistance training, high-intensity strength training and no exercise. The authors demonstrated that each form of exercise significantly reduced depression scale scores compared with those in the control group, in which average depression scale scores increased. The study concluded that the physical activity program effectively improved inmates’ mood and anxiety and overall mental health [21].

In a pilot study published in 2015, Mannocci et al. [22] assessed in the multicentric cross-sectional study the association between physical activity and quality of life among Italian inmates. Inmates from eight prisons compiled a questionnaire. The Metabolic Equivalent of Task was used to measure inmates’ weekly physical activity levels. The authors found a positive association between the quality of life and level of physical activity. Moreover, they demonstrated that the years of detention and age were important aspects of the overall quality of life assessment. The time spent on exercise positively correlated with age and years spent in prison. Inmates with long-term sentences and older individuals feel the need to organize interests/activities to improve how to spend their time and achieve better life satisfaction.

In 2016, a double case study by Amtmann and Kukay [47] examined the effects of fitness coaching on two juveniles at a youth detention facility in Southwest Montan. After the eight-week program, both participants made fitness improvements, and both perceived positive effects on the self-concept and overall sense of well-being from participating in this program.

More recently, Bueno-Antequera et al. [48] evaluated the effects of a 12-week intervention combining aerobic and strength exercises of moderate-to-high intensity in 41 prison inmates with a psychiatric disorder. The authors established that the exercise program led to substantial benefits in cardiorespiratory fitness, upper-body strength, and anthropometric measures.

In 2018, Mohan and coworkers [49], in a systematic review, examined the interventions aimed to improve the cardiovascular health-related factors or behaviors among inmates during imprisonment. They concluded that supervised physical activity improved determining factors such as blood pressure and cardiovascular issues and modified health factors or behaviors of prisoners’ cardiovascular health during incarceration. Moreover, Sfendla et al. [50] analyzed the effect of ten weeks of yoga practice on the mental health profile in prisoners (152 volunteer participants). Participants in this study were randomly placed in either of two groups: to participate in a weekly 90-min yoga class (yoga group) or a weekly 90-min free-choice physical exercise (control group). Results demonstrated that yoga practice improved all primary symptoms when comparing with the control group.

In the same year, Wangmo [51] and coworkers studied the perception of 35 old Swiss inmates (aged 50 or older) of nutritional diets and adequate exercise when incarcerated. The project was part of a larger one that included Switzerland and two other European countries and aimed to investigate aging prisoners’ health care. Study participants were recruited from 12 prisons in Switzerland and were aged 50 or older. In total, 35 older prisoners participated in this study. The mean age was 61 years (range 51–75 years); 30 participants were male and five were female. Regarding exercise and PA prisoners, they did not exercise mostly because they did not wish to or were not healthy enough. A few of them felt no need to exercise, since they were working all day in prison.

Recently, Swiss prisons, especially in the French-speaking part, have been overcrowding [70]. Nevertheless, the mainstem of this correctional system remains the value of the prisoner. Therefore, prison conditions—especially regarding medical treatments—should be similar or equivalent to those provided for the general population.

In 2019, Sanchez-Lastra analyzed in a systematic review the effectiveness of exercise training programs performed by inmates, definitely demonstrating that prison-based exercise programs constitute a feasible and useful strategy for improving the physical and mental health status of prisoners [7].

One year later, Legrand et al. [52] used an interval exercise training intervention to decrease anxiety symptoms in first-time prisoners. 36 first-time prisoners with elevated anxiety symptoms were enrolled in this study and randomly divided into two groups: Interval exercise training (IET; *n* = 20) or no intervention (waiting-list; *n* = 17). Prisoners in the IET intervention had to exercise three times per week (40 min per session). The results demonstrated that the IET intervention group had a significant decrease in anxiety symptoms compared to prisoners in the control group.

## 4. Discussion

Regular PA is proven to help prevent and manage non-communicable diseases such as heart disease, stroke, diabetes, and several oncologic malignancies. Regardless of age, it also helps prevent hypertension, maintains healthy body weight, and can improve mental health, quality of life, and well-being. In 2010, WHO developed the Global Recommendations on Physical Activity for Health for young people and adults that was further revised in 2018.

The percentage of old prisoners is increasing as well as aging in Western countries. Moreover, the number of female inmates is also increasing worldwide. Prisoners also present a higher burden of diseases with a higher risk of chronic pathologies than the general population and more inadequate healthcare. PA plays a major role as a protective factor towards pathologies reported by the prison population and should be considered in all prisoner populations to ameliorate the conditions outlined above. According to WHO, the prescription for PA in older people does not differ from younger adults, males, and females; in a weekly exercise session of 150 min of moderate-intensity (or 75 min of vigorous-intensity), they include a minimum of 10 min duration of aerobic exercise [71]. In addition, strength or resistance exercises should also be performed at least twice a week. Prisoners often are not able to affect their cardiovascular “maybe-modifiable “and “modifiable” risk factors (as defined by Lennon et al. [3]) as well as improve their healthy habits and lifestyles.

Although PA is a powerful tool for cardiac protection and secondary prevention, it is often under-prescribed for people with cardiac comorbidities due to safety concerns. The New York Heart Association has found no evidence that exercise might increase the risk of all-cause death in either the short or long term for patients with stable CVD, so prisoners may safely take advantage of the lower cardiovascular risk factors derived from moderate- and high-intensity exercise programs.

Finally, in cardiac rehabilitation programs, the cardiovascular risk factor seems to lower after moderate- and high-intensity exercise in patients suffering from CVD.

## 5. Conclusions

Here, we aimed to investigate the health conditions in prisoners compared to the general population. Additionally, we tried to assess whether or not physical activity and exercise programs could be a valuable tool to prevent CVD in inmates and improve their physical and mental health. According to the data acquired, the vast majority of prisoners suffer from serious mental illness and cardiovascular diseases or both.

Unfortunately, with rare exception, a paucity of funding for prisoner healthcare and well-being exists across prison systems worldwide. Another important issue is the feasibility of PA programs in the various countries and related jail institutions, due to the difficulties to implement such programs due the lack of related policies and perceived “waste” of resources in the case of interventions aimed to improve the “well-being” of prisoners. Although prisoners’ health care and their well-being certainly might not be a priority for the government almost worldwide, the promotion of PA among inmates should be a prescribed intervention. We believe that physical activity and exercise training might be an effective public health strategy able to improve the health status of the prison population, decreasing the risk of cardiovascular events and increasing psychological well-being in inmates, therefore providing substantial long-term financial benefits. Therefore, given the low cost of basic exercise programs and their significant health benefits, PA should be adopted as a public health strategy for prisoners.

## Figures and Tables

**Table 1:** Summary table of the studies included in the present systematic literature review and narrative description.

Study Details	Study Design	Target Group	Topic
Hachbardt et al., Cardiovasc Prev 2020 [1]	Research article	Female prisoners	CVD in prison
Wang et al., Journal of the American College of Cardiology 2017 [5]	Review	Old prisoners	CVD in prison
Fazel and Baillargeon, The Lancet 2011 [6]	Review	General prison population	Prisoners health conditions
Sanchez-Lastra et al., Journal of Physical Activity and Health 2019 [7]	Systematic review	General prison population	PA in prison
Battaglia et al., Crim Behav Ment Health 2015 [21]	Research article	General prison population	PA in prison
Mannocci et al., European Journal of Public Health 2018 [22]	Research article	General prison population	PA in prison
Cashin et al., Psychiatric and Mental Health Nursing 2008 [23]	Research article	General prison population	PA in prison
Heidari et al., Br Dent J 2014 [30]	Review	Male prisoners	Prisoners health conditions
Williams et al., J Am Geriatr Soc 2012 [36]	Review	Old prisoners	Prisoners health conditions
Fazel and Danesh, The Lancet 2002 [38]	Systematic review	General prison population	Prisoners health conditions
Condon et al., J Clin Nurs 2007 [39]	Review	General prison population	Prisoners health conditions
Wilper et al., Am J Public Health 2009 [40]	Research article	General prison population	Prisoners health conditions
Butler et al., Australian and New Zealand Journal of Public Health 2004 [41]	Research article	General prison population	Prisoners health conditions
Munday et al., Age and Ageing 2019 [42]	Systematic review and meta-analysis	Old prisoners	Prisoners health conditions
Packham et al., BMJ Open 2020 [43]	Research article	Male prisoners	CVD in prison
Greene et al., Health Justice 2018 [44]	Research article	Old prisoners	Prisoners health conditions
Fazel, Age and Ageing 2001[45]	Research article	Old prisoners	CVD in prison
Plugge et al., Health Promotion International 2009 [46]	Research article	Female prisoners	CVD in prison
Amtmann and Kukay, Journal of Correctional Health Care 2016 [47]	Research article	Young prisoners	PA in prison
Bueno-Antequera et al., Clin Rehabil 2019 [48]	Research article	Male prisoners	PA in prison
Mohan et al., The Journal of Cardiovascular Nursing. 2018 [49]	Systematic review	General prison population	PA in prison
Sfendla et al., Front. Psychiatry 2018 [50]	Research article	General prison population	PA in prison
Wangmo et al., J Correct Health Care 2018 [51]	Review	Old prisoners	PA in prison
Legrand et al., Anxiety, Stress, & Coping 2020 [52]	Research article	General prison population	PA in prison

**Table 2:** Summary of findings of the nine studies evaluating the health conditions in prisoners.

Study Details	Study Design	Target Group	Summary of Findings
Fazel and Baillargeon, The Lancet 2011 [6]	Review	General prison population	The authors discussed the prevalence and risk factors for some of the major physical and psychiatric diseases in prisoners. They highlighted that women, prisoners aged 55 years or older, and juveniles present higher rates of many disorders than other prisoners.
Heidari et al., Br Dent J 2014 [30]	Review	Male prisoners	The authors offered an overview of the general and oral health status of male prisoners. They established that the prison population is relatively young; however, their health status is worse than the general population matched by age. Factors such as smoking, abuse of alcohol or drugs, mental health, and medical comorbidities contribute to this poor health status.
Williams et al., J Am Geriatr Soc 2012 [36]	Review	Old prisoners	The authors provided an overview of aging in the criminal justice system and described how geriatric care models could be adapted to address the mounting older prisoner healthcare crisis.
Fazel and Danesh, The Lancet 2002 [38]	Systematic Review	General prison population	The authors provided a systematic review of surveys on SMI in general prison populations in Western countries. Their results suggest that about 14% in Western countries have SMI, and about 50% of male prisoners and about 20% of female prisoners have antisocial personality disorders.
Condon et al., J Clin Nurs 2007 [39]	Review	General prison population	The authors reviewed the primary healthcare needs of prisoners in England and Wales. They established that the prison population’s health needs are much greater than those of the general population. Prisoners have a higher incidence of SMI and long-standing physical illnesses and disabilities.
Wilper et al., Am J Public Health 2009 [40]	Research article	The data analyzed were from the 2004 Survey of Inmates in State and Federal Correctional Facilities (SISFCF) and the 2002 Survey of Inmates in Local Jails (SILJ)	The authors analyzed the prevalence of chronic illnesses, including mental illness and CVD, in US inmates. Among inmates in federal prisons, state prisons, and local jails, 38.5%, 42.8%, and 38.7%, respectively, suffered from a chronic medical condition. Among prisoners reporting a psychiatric disorder, almost 60% were on a psychiatric medication after admission, whereas 30% suffered from hypertension, and 4% reported prior myocardial infarction.
Butler et al., Australian and New Zealand Journal of Public Health 2004 [41]	Research article	747 men and 167 women in 29 (27 male and two female) correctional centres	The authors described the physical health of the New South Wales prisoner population. Despite the comparatively young population, 81% of women and 65% of men had at least one chronic health condition; 41% of men and 59% of women reported multiple health problems. Moreover, chronic conditions were more prevalent among women prisoners: 37% of women and 28% of men rated their health as either “poor” or “fair” compared to 16% of women and 15% of men in the general NSW general population.
Munday2/26/2021 2:33:00 PM et al., Age and Ageing 2019 [42]	Systematic review and meta-analysis	Old prisoners	The author studied the prevalence of the non-communicable disease in older people in prison. Their analysis provided data on 28 NCDs in 93,862 individuals from prisons in 11 countries. Prisoners over 50 years of age experienced a higher burden of NCD than younger prison and age-matched community peers. Lifestyle, environmental, and societal factors influence health inequality. Pooled prevalence for the most significant NCDs was: cancer 8%, CVD 38%, hypertension 39%, and diabetes 14%.
Greene et al., Health Justice 2018 [44]	Research article	The study compared 238 older jail inmates age 55 or older to 6871 older adults in the national Health and Retirement Study (HRS)	All geriatric conditions were significantly more common in jail-based participants than in HRS participants. Moreover, geriatric conditions are prevalent in older adults in jail at significantly younger ages than those non-incarcerated.

**Table 3:** Summary of the finding of the five studies evaluating the incidence of CVD and CHD in the prison population.

Study Details	Study Design	Target Group	Summary of Findings
Hachbardt et al., Cardiovasc Prev 2020 [1]	Research article	120 female prisoners	The authors evaluated the cardiovascular risk in incarcerated women from a public prison in Brazil. Participants included in the study initially presented a low cardiovascular risk. Nevertheless, the analysis of the anthropometric features revealed an increasing trend in the CV risk.
Wang et al., Journal of the American College of Cardiology 2017 [43]	Review	Old prisoners	The authors reviewed the current status of cardiovascular health of justice-involved populations and established that general risk factors in addition to incarceration-specific factors (exposure to the prison environment, poor diet, or lack of exercise) might increase the risk of CVD.
Packham et al., BMJ Open 2020 [43]	Research article	Male prisoners in 13 prisons. All prisoners eligible for the NHS Healthcheck Program in prison settings were initially enrolled. 1207 prisoners completed a Healthcheck	The authors analyzed the cardiovascular risk profiles in male prisoners in six UK prisons: 1207 prisoners completed a Healthcheck Program, and of those, 12.1% had new significant CVD comorbidity. CVD risk was similar to community levels, but this population was 10 years younger. Moreover, the authors determined high rates of anxiety and depression in this cohort.
Fazel, Age and Ageing 2001 [45]	Research article	Old prisoners aged 60 or older. 203 men from 15 prisons	The authors investigated the health of men aged 60 and over in English and Welsh prisons. They interviewed 203 men from 15 prisons and demonstrated that 85% of the elderly prisoners had one or more major illnesses reported in their medical records; 83% reported at least one chronic illness in the interview. The most common illnesses were psychiatric, cardiovascular, musculoskeletal, and respiratory. Moreover, 35% of the interviewed reported CVD compared to 29% in the community-based elderly men.
Plugge et al., Health Promotion International 2009 [46]	Research article	505 Female prisoners in England	The authors examined the prevalence of five modifiable cardiovascular risk factors in women prisoners on entry to prison and one month after imprisonment. The results showed that women prisoners were at high risk of CVD in the future; 85% smoked cigarettes, 87% were insufficiently active, 86% had inadequate food intake or diet habits, and 30% were overweight or obese. After one month, there were few improvements in risk factors.

**Table 4:** Summary of the finding of the ten studies evaluating the feasibility and the effectiveness of exercise programs in prisoners.

Study Details	Study Design	Target Group	Summary of Findings
Sanchez-Lastra et al., Journal of Physical Activity and Health 2019 [7]	Systematic Review	General prison population	The authors analyzed in a systematic review the effectiveness of exercise training programs performed by inmates. A total of 11 randomized controlled studies were selected. After the intervention, 10 out of the 11 studies reported significant changes in physical and mental health-related variables.
Battaglia et al., Crim Behav Ment Health 2015 [21]	Research article	64 participants; three groups: CRT, HIST and no exercise.	The authors performed a nine-month training program to assess the psychological welfare of 64 prisoners. The authors demonstrated that every type of training program significantly reduced depression scale scores compared with those in the control group.
Mannocci et al., European Journal of Public Health 2018 [22]	Research article	636 volunteers enrolled; 398 included.	In a multicentric cross-sectional study, the authors assessed the association between physical activity and quality among Italian inmates from eight prisons. They established a positive association between the quality of life and the level of physical activity. Moreover, the years of detention and age were important aspects of the overall quality of life assessment. The time spent on exercise positively correlated with age and years spent in prison
Cashin et al., Psychiatric and Mental Health Nursing 2008 [23]	Research article	914 prisoners, 747 male and 167 female	The authors investigated the relationship between the levels of self-reported physical exercise and mental well-being in a cohort of 914 prisoners within New South Wales, Australia. A significant inverse relationship between self-reported exercise in minutes per week and hopelessness was identified.
Amtmann and Kukay, Journal of Correctional Health Care 2016 [47]	Research article	2 male young prisoners ages 16 and 19	In a double case study, the authors examined the effects of fitness coaching on two young prisoners. They demonstrated that after an eight-week program, both participants made fitness improvements, and both perceived positive effects on the self-concept and overall sense of well-being from participating in this program.
Bueno-Antequera et al., Clin Rehabil 2019 [48]	Research article	Male prisoners 41 prisoners with psychiatric disorders; intervention group = 21; control group = 20.	The authors evaluated the feasibility and effects of a 12-week intervention combining aerobic and strength exercises in prison inmates with psychiatric disorders. The exercise program included three weekly sessions of group-based moderate-to-high intensity combined exercises. Despite the high dropout rate, exercise sessions seemed to be effective and improved fitness and anthropometric measures.
Mohan et al., The Journal of Cardiovascular Nursing. 2018 [49]	Systematic Review	General prison population	The authors reviewed the possible interventions aimed at improving cardiovascular health-related factors or behaviors among inmates during imprisonment. They concluded that PA might modify health factors or behaviors of the cardiovascular health of prisoners during incarceration.
Sfendla et al., Front. Psychiatry 2018 [50]	Research article	152 prisoners (133 men; 19 women). Control group= 15; yoga group = 77)	The authors analyzed the effect of 10 weeks of yoga practice on the mental health profile of 152 prisoners and assessed that yoga practice improved all primary symptoms when compared to the control group.
Wangmo et al., J Correct Health Care 2018 [51]	Review	Old prisoners	The authors studied the perception of 35 old Swiss inmates (aged 50 or older) regarding diet and exercise when incarcerated. Prisoners reported that they do not exercise mostly because they do not wish to or are not healthy enough. A few of them felt no need to exercise, since they are working all day in prison.
Legrand et al., Anxiety, Stress, & Coping 2020 [52]	Research article	37 prisoners. IET group = 20; control group = 17).	The authors used an interval exercise training (IET) protocol to decrease anxiety symptoms in first-time prisoners. The results demonstrated that the IET intervention group had a significant decrease in anxiety symptoms compared to prisoners in the control group.

## Abbreviations

CVD	Cardiovascular Disease
CHD	Coronary Heart Disease
PA	Physical Activity
PTSD	Post-traumatic Stress Disorder
WHO	World Health Organization
